# New horizons in modulating the radio-sensitivity of head and neck cancer - 100 years after Warburg’ effect discovery

**DOI:** 10.3389/fonc.2022.908695

**Published:** 2022-12-08

**Authors:** Camil Ciprian Mireștean, Roxana Irina Iancu, Dragoș Petru Teodor Iancu

**Affiliations:** ^1^ Department of Medical Oncology and Radiotherapy, University of Medicine and Pharmacy Craiova, Craiova, Romania; ^2^ Department of Surgery, Railways Clinical Hospital, Iasi, Romania; ^3^ Oral Pathology Department, “Gr.T.Popa” University of Medicine and Pharmacy, Iasi, Romania; ^4^ Department of Clinical Laboratory, St. Spiridon Emergency Hospital, Iasi, Romania; ^5^ Department of Medical Oncology and Radiotherapy, “Gr.T.Popa” University of Medicine and Pharmacy, Iasi, Romania; ^6^ Department of Radiation Oncology, Regional Institute of Oncology, Iasi, Romania

**Keywords:** Warburg effect, head and neck cancers, HNSCC, radio-sensitivity, radio-resistance

## Abstract

Tumor radiation resistance along with chemotherapy resistance is one of the main causes of therapeutic failure of radiotherapy-treated head and neck cancers. 100 years after the discovery of the Warburg effect, a process specific to malignant cells to metabolize glucose especially anaerobically even under normoxia condition, its modulation has become a viable therapeutic target for improving the results of cancer therapies. Improving the radio-sensitivity of head and neck tumors by reversing the Warburg effect can increase the rate of local control and reduce the toxicity associated with irradiation. P53 status can be used as a biomarker in the choice of a single agent strategy (cell respiration inhibition with Metformin) or double inhibition, both of respiration and glycolysis. Targeting of enzymes involved in the Warburg effect, such as Hexokinase-II, are strategies with potential to be applied in clinical practice with radio-sensitizing effect for head and neck squamous cell carcinoma. Even if anti-Warburg therapies tested in clinical trials have been associated with either toxic deaths or a minor clinical benefit, the identification of both potential radio-sensitivity biomarkers and methods of reversing the Warburg effect will play an important role in the radiobiology of head and neck cancers.

## Introduction

Even though it is only the 6th malignancy as a worldwide incidence, head and neck cancers are notable for the high rate of therapeutic failures that leads to a survival of 50-60% at 5 years. Radiation resistance and chemotherapy resistance are decisive factors that lead to this increased rate of unfavorable responses. Identifying biomarkers of radio-resistance but also strategies to overcome this phenomenon are essential strategies in personalizing treatment, escalation or de- escalation from case to case with consequences both in increasing local-regional control and in limiting toxicity. 100 years after its discovery, the Warburg effect returns to the forefront as a key player involved in the radiobiology of head and neck cancers (1–3).

The Warburg effect and its modulation are involved in chemo-resistance and, through acidification, some agents including Paclitaxel can be neutralized by the acidic microenvironment. “Ion trapping mechanism” is the mechanism by which the agent is inactivated by the acidity of the

intracellular environment before reaching the target. The immune response is also inhibited by lactate accumulated in T cells which reduces the secretion of pro-inflammatory cytokines, but acidosis also has a synergistic effect with hypoxia, and glycolysis of stromal cells feeds malignant cells, a factor that promotes proliferation and prevents apoptosis. Thus, the tumor microenvironment is intensely influenced by the Warburg effect and the modulation of metabolism indirectly leads to the regulation of chemo-sensitivity and the antitumor immune response. LDHA has an essential role in Taxol resistance, the Warburg effect being involved in this resistance mechanism. The study by Zhou and collaborators demonstrates this phenomenon of chemo- resistance in connection with the Warburg effect only on breast cancer cells. Muramatsu et al. Identifies LDHA as a possible target for overcoming resistance for Docetaxel in castration − resistant prostate cancer cells. It should be noted that taxanes are part of the platinum double or platinum- taxane-fluorouracil triple combination protocol used in HNSCC induction protocols (4–7).

## Warburg effect and cancer - Mechanism of radio-resistance

A peculiarity of metabolism identified almost a century ago by Warburg, Posener and Negelein is the extremely high level of glycolysis and an increased metabolism of glucose to lactic acid in tumor tissue. Of note is the adaptive capacity identified in the tumor cell that can produce glycolysis without depending on an oxygen source. The concept of destroying tumors by depriving them of energy was proposed in 1927 by Otto Warburg, and the resumption of experiments previously proposed by Carl F. Cori showed significant reductions in glucose levels and an increase in lactic acid level in the blood passed through a tumor (8–10).

Observing different levels (57% versus 2-18%) of glucose consumption in tumor tissue respectively normal tissue, Warburg initially hypothesized that it is consumed by fermentation and respiration, but later rectified the theory by considering impaired respiration as the pathophysiological phenomenon of tumor metabolism. The strategy of targeting the mechanisms of control of tumor metabolism is currently one of the proposed concepts for modulating the sensitivity of tumor cells in cancer treatments. Tumor cells have generally been associated with high levels of lactate accumulation, and cases where the Warburg effect is not linked to high levels of lactate may be explained by its consumption by highly glycolytic fibroblasts that consume the product of tumor cell metabolism. Another possible explanation for the discrepancy between the lactate level and the Warburg phenomenon may be a clearance of lactate in the bloodstream. Thus, elevated lactate levels associated with either intense glycolysis or impaired vascularity or both may be a biomarker of a negative prognosis in cancer (2, 9, 10).

By supporting the glycolysis of stromal cells by tumor metabolism and the production of adenosine triphosphate (ATP) by the tumor cell, the stromal-cancer metabolic coupling occurs, a phenomenon that ultimately leads to tumor proliferation. Pyruvate dehydrogenase kinase (PDH- kinase), Hypoxia-inducible factors (HIFs) and Lactate dehydrogenase A (LDHA) enzymes but also mitochondrial malignant cell dysfunction are factors involved in the glycolytic nature of cancer metabolism. HIFs, LDHA and PDH kinases are up-regulated in cancer even under hypoxic conditions. Tumor hypoxia has been recognized as a factor associated with radiation resistance since the 5th decade of the last century. The mechanism of reducing the toxic effect of oxygen on the DNA strand is one of the most important radiobiological phenomena. Mention should be made

of the effect of oncogenes in the EGFR family that modulate hypoxia-mediated radio-resistance on the alpha-subunit of HIFs and also the reverse, acquired resistance to EGFR tyrosine kinase associated with increased HIF-1α levels HIF-1 modulates tumor resistance by manipulating several signaling pathways downstream, with reprogramming of tumor metabolism, vasculogenesis, and epithelial-mesenchymal transition being the most important events associated with tumor response to irradiation. Activation of the HIF-mediated PI3K/AKT/mTOR pathway alters tumor metabolism, making malignant cells more resistant to anticancer drugs. The von Hippel-Lindau mutated tumor suppressor gene can activate HIF-1 *via* Akt-kinase and the PI3K/AKT/mTOR pathway (11–14).

A less known mechanism is that involved in the secretion of cytokines by irradiated tumors, inhibiting apoptosis in endothelial cells and thus reducing treatment-induced vascular damage. HIF-1 is involved in this mechanism. The increase in radiation resistance by methods other than manipulating the Warburg effect but involving HIF1 demonstrates the involvement of mediators of tumor metabolism and other mechanisms of radiation resistance, making HIF-1 an attractive therapeutic target (15).

LDHA is the enzyme with the role of transforming lactate into pyruvate and vice versa, maintaining the NAD+/NADH ratio in physiological metabolism, but it is also associated with a negative prognosis, being indirectly a promotion of tumor growth and metastasis due to the acidification of the tumor microenvironment. Knocking down of LDHA can induce apoptosis and stop the growth of tumor cells. Thus, LDHA had the potential to regulate tumor metabolism, control apoptosis and cell proliferation. Assessed on prostate cancer cell lines LDHA has been validated as a biomarker of glycolysis-associated radio-resistance in cancer cells. Knockdown of LDHA small interfering RNA (siRNA), but also inhibition of LDHA by inhibitory agents such as FX11 or Urolithyn M6, a metabolite produced by gut microbiota, are also possible indirect ways of controlling cellular radio-sensitivity by targeting the Warburg phenomenon. FX11 has demonstrated the ability to inhibit LDHA and thus the effect of Warburg in pancreatic cancer cells only in the presence of mutant p53, but the high proportion of p53 mutant cells in most malignancies makes this a feasible therapeutic option (16–18).

Monocarboxylate transporters (MCTs) that have the value of membrane proteins act as carriers for lactate. The elimination of lactate from the cell is essential, the accumulation of this product of the excess Warburg phenomenon can lead to the death of the malignant cell. Named by Pivovarova and co-workers for the “sweet tooth” of cancer, the Warburg effect is aided by the glucose transporter and inhibition of mono-carboxylate transporters (MCTs) can help cellular acidification. Inhibition of MCT1 in cells that express only this transporter can lower intracellular pH and inhibit cell growth by blocking the cell in the G1 phase of the cycle. A transient hyperglycemic-clamp associated with proton export inhibitors is proposed by the authors as an anti-warburg effect and implicitly antineoplastic. AZD3965, a pyrrole pyrimidine derivative is proposed as anti-MCT1 therapy, having demonstrated tumor suppressor effect on breast cancer cell lines. MCT4 is thought to be induced by metastasis-enhancing mitochondrial NADH dehydrogenase gene mutations in lung cancer and is considered another therapeutic target. The class of inhibitors called syrosingopins, derived from a natural alkali (reserpine) is considered active on both MCT1 and MCT4. Loss of NAD + regeneration capacity by the combination of metformin and syrosingopine aims to double block MCT1 and MCT4 can lead to glycolytic

inhibition, ATP depletion and ultimately cancer cell death. Double-blocked MCT1 and MCT4 are considered 60 times more effective than blockade of a single glucose transporter (19–21).

TKL1 was considered a proto-oncogene, its overexpression being correlated with tumor growth, induction of glycolysis, lactate production and glucose consumption, but also with tumor progression. In head and neck cancers TKTL1 has been associated with carcinogenesis by incressead aerobic glycolysis and HIF1alpha stabilization. TKTL1 protein expression was correlated with an unfavorable prognosis for invasive tumors of the colon and urothelium. TKTL1 is proposed as a therapeutic target for oxygen-independent glucose metabolism, but also for lactate-based matrix degradation. Thus, targeting the Warburg effect with anti-transketolase therapies may underlie the individualization of antineoplastic therapy, by inhibiting invasion and metastasis (22–25).

## Warburg effect and chemo-resistance - Therapeutic synergies in HNSCC

Chemotherapy is part of HNSCC’s multimodal treatment, whether it is administered concurrently with irradiation or as an induction treatment. Cisplatin based chemotherapy is currently the therapeutic standard in combination with radiation therapy given weekly, or every 3 weeks. The synergistic effect of chemotherapy and radiation therapy is attributed to radio- sensitization, a phenomenon based on the formation of DNA adducts that prevent the repair of radiation-induced lesions and lead the malignant cell to apoptosis. Taxanes, 5-Fluorouracil and platinum salts but also Gemcitabine are also part of the induction protocols. As a radio-sensitizer, Cisplatin is a key player in terms of radio-sensitivity, and Warburg-mediated chemo-resistance is indirectly involved in the response to multimodal treatment. Synergistic cooperation is reported between GLUT1 inhibitors and chemotherapy, with the effect of radio-sensitizing and potentiating apoptosis in squamous cell carcinoma of the mouth and larynx. Wang et al. GLUT1 inhibitors have been reported to be associated with increased chemo-sensitivity to Cisplatin (2, 26–28).

Overexpression of p21-activated kinase 2 (PAK2) is frequently identified in head and neck cancers, with a positive correlation of PAK2 with worst outcomes in this type of cancer. The correlation of PAK2 expression with c-Myc-dependent pyruvate kinase M2 (PKM-2) overexpression makes the PAK2- c-Myc-PKM2 axis a key player in the regulation of cell proliferation, aerobic glycolysis and chemoresistance. Targeting this pathway would thus be a viable strategy to restore chemosensitivity, consequently having the effect of limiting cell proliferation. PAK2-c-Myc- PKM2 is considered to have a key role in oncogenesis but also in radio-resistance of head and neck cancers *via* regulating Warburg effect. By activating AMPK and inhibiting mTOR and HIF-1α, Metformin in combination with 5-Fluorouracil increases the tumor response to chemotherapy by regulating the enzymes involved in the Warburg effect in human oral squamous cell carcinoma (4–7, 28–32).

Radiotherapy in combination with chemotherapy, as an adjunct treatment administered after surgery or as a definitive treatment for locally advanced cases is a backbone of the multidisciplinary approach to head and neck cancers, especially in the locally advanced stage. Different sensitivity to irradiation is a major cause of the different response to standard treatment and implicitly radio-resistance is associated with therapeutic failure (32).

Tumor hypoxia is a well-known phenomenon and is frequently associated with the concept of radio-resistance and therapeutic failure. It is considered that approximately 90% of solid tumors

have lower average oxygen concentration values than normal values (40-60 mmHg), with tumor sub-volumes with concentrations <2.5 mmHg. The association of hypoxic cells with radio- resistance and implicitly with therapeutic failures, makes the identification of strategies for overcoming tumor radio-resistance a priority. Fractional modulation, beyond the standard of “2Gy/fraction” is based on radiobiological concepts based on the linear quadratic model (LQ) and cell survival curves. The concept of hypo-fractionation can limit the re-oxygenation capacity of tumor cells by delivering the total dose in fewer fractions. Spatial identification of hypoxic sub- volumes and administration of a radiation dose boost on these areas of the tumor overlaps with the concept of “biological dose painting”. The combination of anatomical and topographic data of the tumor with metabolic parameters leads to an improvement in the distribution of radiation doses by escalating the dose in regions of possible radioresistance, thus increasing the possibility of obtaining a local tumor control. However, the toxicities associated with high doses of radiation and the risk of severe side effects associated with it suggest the use of chemical radio-sensitizers to increase tumor control rates without requiring dose escalation. Targeting tumor metabolism and implicitly the Warburg effect is part of the precision medicine concept and is a current topic almost 100 years after the discovery of the Warburg effect. The effect of improving the immune response of the host after hypo-fractionated irradiation is a research direction for the identification of a new possible application of radiotherapy. However, there are many unknowns, the balance between immunogenic and immunosuppressive mechanisms being in a fragile balance (29, 32–36).

Ohasi et al. identify in HNSCC the lactic acid secreted by tumor cells as a mediator of immunosuppression, with the role of converting macrophages into M2 macrophage, involved in the reduction of the inflammatory response, angiogenesis and tissue remodeling. M2 is also involved in reducing the amplitude of the immune response, thus promoting cancer progression. Positron emission tomography/computed tomography (PET/CT) is proposed to identify the Warburg effect, the polarization of M2 macrophages may be associated with increased glucose uptake. The Warburg phenomenon induces an immunosuppressive environment by polarizing M2 macrophages in HNSCC (37).

**Figure 1:**
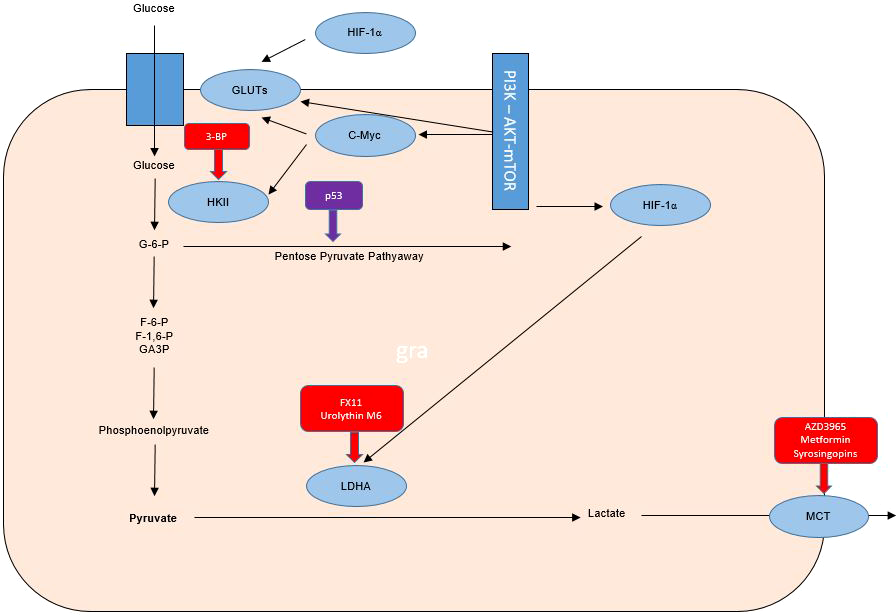
Warburg effect in tumor cells and therapeutic targets for radiosensibility and chemosensibility modulation in head and neck cancers.

The mutant TP53 gene is known to be implicated in the adverse course of cancer. In HNSCC it is known that patients with p53 wild type have a favorable evolution compared to those with mutant p53. The high rate (75 to 85%) of p53 mutations in HNSCC not associated with human papilloma virus (HPV) infection justifies the involvement of p53 in the Warburg effect. Wilkie et al. demonstrate the correlation between loss of p53 function and the Warburg effect in HNSCC. Cancer cells that lose function, either through mutations or mediated down-regulation, are becoming more likely to use the glycolysis pathway, and the authors hypothesize that glycolysis inhibitors may be anti-radio-resistance agents by targeting the Warburg effect (38, 39) (**Figure 1**).

Anti-metabolic strategies based on the p53 mutation in order to overcome radio-resistance included the assessment of radiation sensitivity and the assessment of glycolytic flux and mitochondrial respiration on HNSCCN cell lines expressing p53 mutant and wild type. Consistent with literature and the experiment of Sandulache et al. demonstrated increased radio-resistance associated with mutant p53. Only mutant p53-expressing cells responded to inhibition of glycolysis, with mitochondrial reserves of wild-type p53 cells being considered the cause of the unbalanced response to glycolysis inhibitors. Inhibition of respiration with Metformin and simultaneous inhibition of glycolysis increased the radio-sensitivity of p53 wild type cells. The

authors conclude that p53 is a valid biomarker that guides the radio-sensitization strategy towards Warburg effect inhibitors for mutant p53-expressing cancers and towards a combination of glycolysis and respiratory inhibitors for wild-type p53 cancers (40, 41).

Hexokinase-II (HK-II) is the essential enzyme in limiting the rate of glycolysis and is a catalyst for the conversion of glucose to glucose-6-phosphate. Inhibition of HK-II expression may increase the radio-sensitivity of laryngeal cancer cells by reprogramming tumor metabolism. The radio-resistance modulation effect of small interfering RNA (siRNA) on Tu212 laryngeal cancer cells was quantified in the study by Chen et al. evaluating both cell survival, proliferation, apoptosis and cell cycle, but also oxygen consumption, lactic acid production, glucose consumption, ATP levels and glycolysis regulatory enzymes. The association of irradiation with siRNA-mediated HK-II inhibition led to increased radio-sensitivity of Tu212 cells by 2 mechanisms (inhibition of glycolysis and reduction of oxidative phosphorylation). HK-II inhibition is considered a therapeutic method with the potential to increase the radio-sensitivity of laryngeal cancer, thus demonstrating the major role of the Warburg effect in radio-resistance of this type of cancer (37, 38) (**Figure 1**).

TKTL1 protein expression has been linked to an unfavorable prognosis of invasive tumors of the colon and urothelium. TKTL1 is proposed as a therapeutic target for oxygen-independent glucose metabolism but also for lactate-based matrix degradation. Thus, targeting the Warburg effect with anti-transketolase therapies may underlie the individualization of antineoplastic therapy, by inhibiting invasion and metastasis. The administration of anti-Warburg effect therapies in clinical practice is a promising strategy, but initial estimates have been contradicted by the results of the application of these therapies in clinical practice, with initial studies being associated with toxic deaths despite a satisfactory tumor response. Understanding the pharmacokinetics of 3- BP is necessary, there is the hypothesis of a major risk of cellular toxicity, especially in highly ATP-/mitochondrion-dependent cases. Also, 3-BP is reported to be associated with numerous problems in clinical practice through toxic effects (sensed by venous burn), but also rapid inactivation in glutathione-rich tumors and the impossibility of crossing the blood brain barrier. The major impact on the anticancer treatment of this metabolism inhibitor is associated with the last 8 years of research by Professor André Goffeau, but also with serious cases of violation of medical ethics by the use outside of clinical trials by alternative medical practitioners. An overdose of 3-BP was associated in this case with 2 deaths out of 3 cases in which it was administered. However, an association of antimycin and menadione with 3-BP can, through the mechanism of unbalancing mitochondrial ROS production, maintain the toxic effect on glioblastoma cells even if small doses of 3-BP are used to limit toxicity. Chemotherapeutic agents such as cisplatin, doxorubicin, daunorubicin, 5-fluorouracil and modern chemotherapeutic treatment strategies including conjugates, wafer, liposomal nanoparticles and aerosol were proposed by Fan et al. for a synergistic chemotherapy-3-Bromopyruvate (3-BP) combination in clinical practice. 3-BP, one of the most promising anti-metabolic drugs, has been associated with 2 toxic deaths in young patients with advanced hepatocellular carcinoma and melanoma, but is also limited by the inability to cross the blood-brain barrier and the burning sensation of the vein. Other substances including Resveratrol as a GLUT1 inhibitor that have been tested in clinical trials have not been associated with severe toxic effects but have been shown to have reduced antitumor bioactivity possibly due to poor solubility (42–50) (**Figure 1**).

## Conclusions

100 years after its discovery, the Warburg effect gains new value in modulating tumor radio-sensitivity. The topic is of particular interest for head and neck cancers, given the high rate of therapeutic failures associated with tumor radio-resistance, but also the potential to personalize therapy, improve local control and reduce the rate of toxicity associated with treatment. P53 status can be used as a biomarker in the choice of a single agent approach (cell respiration inhibition with Metformin) or double inhibition, both of respiration and glycolysis. Targeting of enzymes involved in the Warburg effect, such as Hexokinase-II, are strategies with potential to be applied in clinical practice with radio-sensitizing effect for HNSCC. Even if anti-Warburg therapies tested in clinical trials have been associated with either toxic deaths or a minor clinical benefit, the identification of both potential radio-sensitivity biomarkers and of methods for reversing the Warburg effect will open hew horizons for clinical radiobiology of head and neck cancers. Sensitivity to chemotherapy is also influenced by the Warburg effect and the effect of simulated radio-sensitization and chemo- sensitization may generate a synergistic therapeutic effect of strategies for manipulating tumor metabolism.

## Author contributions

All authors listed have made a substantial, direct, and intellectual contribution to the work, and approved it for publication.
